# Instrumental Evaluation of COVID-19 Related Dysautonomia in Non-Critically-Ill Patients: An Observational, Cross-Sectional Study

**DOI:** 10.3390/jcm10245861

**Published:** 2021-12-14

**Authors:** Simone Bellavia, Irene Scala, Marco Luigetti, Valerio Brunetti, Maurizio Gabrielli, Lorenzo Zileri Dal Verme, Serenella Servidei, Paolo Calabresi, Giovanni Frisullo, Giacomo Della Marca

**Affiliations:** 1 School of Medicine and Surgery, Catholic University of Sacred Heart, Largo Francesco Vito, 1, 00168 Rome, Italy; bellavia.sim@gmail.com (S.B.); irene.scala92@gmail.com (I.S.); serenella.servidei@policlinicogemelli.it (S.S.); paolo.calabresi@policlinicogemelli.it (P.C.); giacomo.dellamarca@policlinicogemelli.it (G.D.M.); 2 Dipartimento di Scienze dell’Invecchiamento, Neurologiche, Ortopediche e della Testa-Collo, Fondazione Policlinico Universitario Agostino Gemelli IRCCS, 00168 Rome, Italy; valerio.brunetti@guest.policlinicogemelli.it (V.B.); giovanni.frisullo@policlinicogemelli.it (G.F.); 3 Department of Emergency, Fondazione Policlinico Universitario Agostino Gemelli IRCCS, Università Cattolica del Sacro Cuore, Largo Gemelli 8, 00168 Rome, Italy; maurizio.gabrielli@policlinicogemelli.it; 4 Digestive Disease Center, Fondazione Policlinico Universitario Agostino Gemelli IRCCS, 00168 Rome, Italy; lorenzo.zileridalverme@policlinicogemelli.it

**Keywords:** SARS-CoV-2, COVID-19, autonomic dysfunction, dysautonomia, Sudoscan, automated pupillometry, heart rate variability, pulse transit time

## Abstract

Coronavirus disease-19 (COVID-19) is a predominantly respiratory syndrome. Growing reports about a SARS-CoV-2 neurological involvement, including autonomic dysfunction (AD), have been reported, mostly in critically-ill patients, or in the long-COVID syndrome. In this observational, cross-sectional study, we investigated the prevalence of AD in 20 non-critically-ill COVID-19 patients (COVID+ group) in the acute phase of the disease through a composite instrumental evaluation consisting of Sudoscan, automated pupillometry, heart rate variability (HRV), and pulse transit time (PTT). All the parameters were compared to a control group of 20 healthy volunteers (COVID− group). COVID+ group presented higher values of pupillary dilatation velocities, and baseline pupil diameter than COVID− subjects. Moreover, COVID+ patients presented a higher incidence of feet sudomotor dysfunction than COVID− group. No significant differences emerged in HRV and PTT parameters between groups. In this study we observed the occurrence of autonomic dysfunction in the early stage of the disease.

## 1. Introduction

Severe Acute Respiratory Syndrome Coronavirus 2 (SARS-CoV-2), a novel coronavirus isolated in China in December 2019 [1], is the pathogenic agent of coronavirus infectious disease (COVID-19), that rapidly spread, in only a few months, across the world. Although lung and respiratory tract symptoms are prevalent and often severe [2], growing evidence is emerging about the neurological involvement of SARS-CoV-2, which could occur in one third of patients with acute COVID-19 [3]. SARS-CoV-2-related neuronal damage could be induced both by a direct cellular invasion [4,5], mediated by the linkage between the virus spike protein and the endothelial acetylcholine receptor [6], or by a cytokine mediated dis-immune mechanism [7,8,9,10].

Among neurological manifestations, recent evidence of autonomic dysfunction (AD) has been reported in the context of the long-COVID syndrome [11,12], but extensive data are lacking about the early onset of dysautonomia in acute COVID-19, especially in non-critically-ill patients. In a study conducted on a bigger cohort of patients which included the cohort of this study, (Scala and Bellavia et al., submitted) we found a high prevalence of orthostatic hypotension and symptoms of dysautonomia, as assessed by COMPASS31 [13].

To date, several tools, such as automated pupillometry (AP), Sudoscan, heart rate variability (HRV), and pulse transit time (PTT), have been employed in order to obtain an indirect evaluation of the autonomic nervous system (ANS). AP is an economical, safe, and clinically validated method to investigate the pupillary light reflex (PLR) [14], defined as the constriction of the pupil consequent to an increase in the illumination of the retina [15]. In fact, ANS, based mainly on ambient light level, continuously adapts the pupil diameter size in order to obtain the best retinal image quality by activating or inhibiting the sphincter and dilator muscles. The AP-based evaluation of PLR to assess the presence of AD has been employed in several medical conditions [16,17,18].

Sudoscan (Impeto Medical, Paris, France) is a simple, standardized, non-invasive tool, able to assess the sudomotor function through measurement of sweat chloride concentrations using reverse iontophoresis and chronoamperometry [19,20]. Sudomotor function is an indirect index of sympathetic cholinergic non-myelinated C-fibers activity, since sweat glands lack parasympathetic innervation. Several authors have reported the efficacy of Sudoscan in detecting AD in some neurological diseases, such as diabetes [21], mitochondrial diseases [22], amyloidosis [23], and narcolepsy [24].

HRV represents the variation over time of the period between consecutive heartbeat, mostly dependent on the extrinsic regulation of the heart rate (HR) mediated by the ANS branches. Thanks to its absolute non-invasiveness and ease of use, HRV has rapidly become a standardized, non-invasive, and widely used tool for assessing the status of the cardiovascular sympatho-vagal balance [25,26]. Through the quantification of the high-frequency (HF) and low-frequency (LF) oscillatory components of the heart rate, HRV is able to discriminate the respective role of the ortho-sympathetic and the parasympathetic components of the ANS [25].

PTT is the time employed by the pulse pression waveform to travel along two different arterial sites [27]. This parameter is an indirect measure of peripheral vasoconstriction and, consequently, it can reflect the sympathetic/parasympathetic balance [28].

In our previous study (Scala and Bellavia et al., in press), we observed orthostatic hypotension in a high percentage of COVID-19 patients. To better characterize the AD features in the same cohort of patients, we performed a composite, instrumental analysis of AP, Sudoscan, HRV and PTT.

## 2. Materials and Methods

### 2.1. Study Design and Population

The study cohort was a subgroup of the one described in our previous paper (Scala and Bellavia, et al., submitted). The study design was single-center, prospective, and cross-sectional. Consecutive patients affected by COVID-19 (COVID+ group) admitted to the COVID-19 sub-intensive care unit or to the COVID-19 regular ward of IRCCS Fondazione Policlinico Agostino Gemelli in Rome were enrolled. Enrolment period was from 1 May 2021 to 31 July 2021.

Inclusion criteria included: (1) active SARS-CoV-2 infection at the time of recruitment confirmed by a reverse transcriptase-polymerase chain reaction (RT-PCR) test for SARS-CoV-2, (2) adult age (≥18 years), and (3) ability to sign informed consent. Exclusion criteria were: (1) inability to maintain orthostatism for the time needed to perform Sudoscan, (2) diabetes, (3) atrial fibrillation, ventricular bigeminy, or trigeminy during the observation period, (4) non-invasive respiratory support, (5) major vision loss, (6) cognitive impairment, (7) language barrier, (8) abnormal neurological examination, and (9) disturbances of state of consciousness.

The study conformed the principles of the 1964 Declaration of Helsinki and its later amendments. The research protocol was approved by the etic committee of our institution (Comitato Etico of Fondazione Policlinico Universitario “A Gemelli” IRCCS—Rome, prot. number 0014686/21). Written informed consent was obtained from the patients at the time of hospital admission. 

### 2.2. Control Group

Control group (COVID−) was composed of 20 subjects enrolled among healthy volunteers (refer to Scala and Bellavia, et al., in press).

Inclusion criteria for COVID− were (1) nasal swab PCR test negative for SARS-CoV-2 infection performed within 48 h, (2) adult age, and (3) ability to sign informed consent. Exclusion criteria were: (1) previous COVID-19, (2) language barrier, (3) abnormal neurological examination, (4) fever, (5) ongoing infections, (6) cognitive impairment or disturbances of state of consciousness, and (7) major vision loss.

The control group was matched with the COVID+ group for sex, age, and Body Mass Index (BMI, kg/m^2^). One control subject was enrolled for each case.

**Figure 1:**
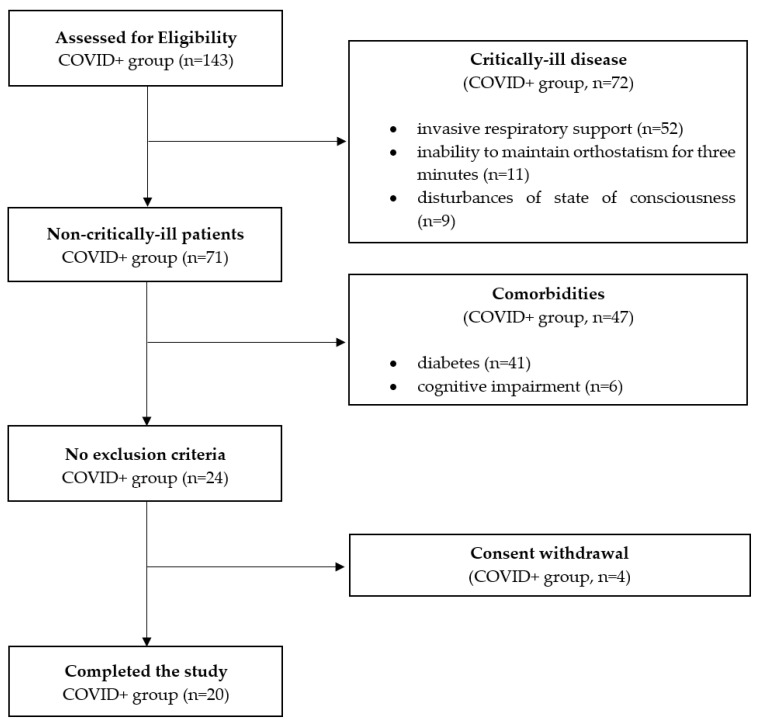
Flow diagram of the study (COVID+ group). Abbreviations: COVID, Coronavirus infectious disease.

The enrollment process is depicted in Figure 1.

### 2.3. Automated Pupillometry

Quantitative automated PLR was measured in both eyes for each member of the study by means of NPi-200 (NeurOptics, Irvine, CA, USA), a handheld portable device composed of an infrared camera that integrates a calibrated light stimulation of fixed intensity (1000 lux) and duration (0.8 s) to provide rapid measurements of PLR parameters regardless of ambient lighting condition. NPi-200 stores repeated video images at >30 frames per second for 3.2 s to calculate PLR parameters. Variables recorded consist of baseline pupil diameter (BPD), minimum pupil diameter at peak of constriction, reflex latency (RL, i.e., the time delay between the light stimulus and the onset of PLR), average constriction velocity (CV), maximum constriction velocity (MCV), average dilatation velocity (DV), constriction index (CI, i.e., BPD minus minimum size divided by BPD), neuro-pupillary index (NPi, i.e., a composite parameter, indicative of pupil reactivity, derived from the combination of all of the previous parameters), and date and time of measurement. Results for each examination are then rapidly displayed on a liquid crystal display [29,30]. 

Data were then reported on an Excel file and mean values of the two eyes’ parameters were calculated and considered for further analysis. Absolute constriction amplitude (ACA) was obtained by subtracting minimum pupil diameter from BPD.

For each subject, AP was performed first in the right eye, while the patients were lying in bed.

**Figure 2:**
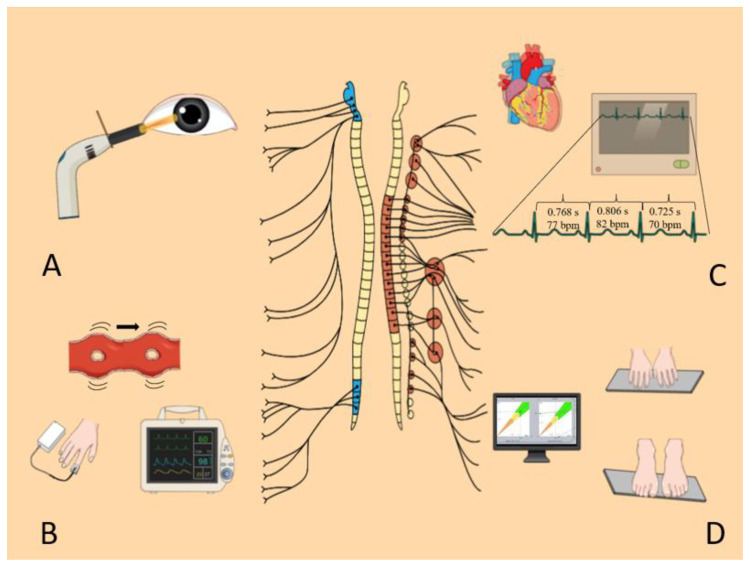
Overview of the techniques used in the study. Panel (**A**) Automated pupillometry (AP) measures the pupil’s light response (PLR) parameters, allowing an indirect evaluation of sympathetic/parasympathetic system modulation. Panel (**B**) Pulse transit time (PTT) was measured by means of a pulse oximeter placed on the index. PTT corresponded to the time lapse between the detection of the R peak on EKG and the peaks in the pulse oximetric signals. Panel (**C**) Heart rate variability (HRV) measurements are based on the variability in the interval between consecutive R peaks as well as the oscillation between consecutive instantaneous heart rates. Panel (**D**) Sudoscan stimulates sweat glands with low voltage direct current and induces an electrochemical reaction between sweat chloride and the stainless-steel electrodes. The parameter obtained is the electrochemical skin conductance (ESC), indirectly evaluating small fibers’ function.

As extensively reported in the literature, an NPi ≥ 3 was considered normal [30] (Figure 2A).

### 2.4. Sudoscan

Sudoscan is composed of a computer connected to two sets of stainless steel electrodes, on which the subject’s hand and feet are placed [19]. By stimulating sweat glands with low voltage direct current (<4 mV), the machine induces an electrochemical reaction between sweat chloride and the stainless-steel electrodes. The parameter obtained is the electrochemical skin conductance (ESC), derived by the ratio between the measured current and the voltage utilized, expressed in microSiemens (µS) [31].

For this study, all COVID+ and COVID− subjects put their hands and feet on the electrodes for 3 min, while maintaining orthostatic position. Mean scores of both hand and feet, and the individual values of each limb, calculated automatically by the machine, were used for further analysis.

An ESC ≥ 70 µS for feet and ≥ 60 µS for hands was considered normal. ESCs of between 50–70 µS (feet) and 40–60 µS (hands) were considered an index of moderate sudori-motor dysfunction, while an ESC < 50 µS (feet) and < 40 µS (hands) were defined as a marker of sever sudomotor dysfunction, as proposed by previous authors [32,33] (Figure 2D).

### 2.5. Heart Rate Variability

All the members of the COVID+ and COVID− groups underwent a bipolar 10 min electrocardiogram (EKG) in supine position, and then 3 min EKG during active standing. Sampling rate was 256 Hz. The two electrodes were placed according to the Lead II modified derivation (the negative electrode was positioned upon the right clavicula and the positive one on the left lower torso). Artifact rejection was performed visually; periods of EKG recordings characterized by ventricular extrasystoles, movements, muscular artifacts, or other artifacts were excluded from the analysis. Dedicated software (SleepView, Medcare Automation B.V., Amsterdam, The Netherlands) recognized the individual electrocardiographic R wave peaks and calculated the R–R intervals (tachogram). Successively, the tachogram was converted into an ASCII file and analyzed by means of a dedicated freeware (HRV Analysis Software, Biomedical Signal analysis Group, Dept. of Applied Physics, University of Kuopio, Kuopio, Finland) [34].

HRV analysis was performed in time-domain for mean HR, mean RR, SDNN, SDANN, NN50, and RMSSD. In the frequency-domain, HRV was analyzed by means of the parametric Autoregressive Model which allows an accurate estimation of Power Spectral Density when analyzing short time intervals during which the signal is supposed to remain stationary [25,35]. The frequency bands considered were low frequency (LF, 0.04–0.15 Hz) and high frequency (HF, 0.15–0.4 Hz) ones. The power of LF and HF bands was expressed in absolute values (ms^2^).

A detailed description of HRV analysis, standards of measurement, physiological interpretation, and clinical use is available in the report of the taskforce of the European Society of Cardiology and the North American Society of pacing and electrophysiology [25,35] (Figure 2C).

### 2.6. Pulse Transit Time

Peripheral hemoglobin saturation was measured by means of a pulse oximeter placed on the index, connected to a computer. A 10 min registration in lying position and a subsequent 3 min recording in orthostatism were performed contemporarily to EKG registration. As previously described in the literature, we identified the starting point with the R peaks of the EKG, which correspond approximately to the opening of the aortic valve, and the terminal point as the pulse pression arrival points detected by a finger pulse oximeter [36]. The traces registration, the detection of the R-peaks in the EKG trace, the peaks in the pulse oximetric signals, and the calculation of the time-interval between the two markers (i.e., PTT) were performed by dedicated software (Rembrandt SleepView, Medcare). Sampling rate was 256 Hz. Subsequently, a visual analysis of the pulse oximetric traces was performed, and PTTs derived by pulse oximetric waves with visible artifacts were manually excluded from the analysis (Figure 2B).

### 2.7. Statistical Analysis

A normality test (i.e., Shapiro-Wilk test) was performed to assess variables distribution. Therefore, continuous variables were summarized as mean ± standard deviation (SD) or as median and interquartile range (IQR) according, respectively, to their normal or not-normal distribution. Categorical variables were expressed as number (*n*) and percentage (%).

Comparisons between the COVID+ and COVID− groups in terms of AP and Sudoscan parameters were performed by means of t-student test for continuous variables following a normal distribution, and through Mann-Whitney U-test for not-normal distributed variables. ESC parameters were dichotomized using the cut-off of 70 µS for feet and 60 µS for hands. For categorical variables we adopted Pearson’s chi-square (χ^2^). 

Finally, in order to test the effect of the interaction between COVID-19 and the particular position assumed (COVID * position) on HRV and PTT parameters, the comparison was performed by means of two-way analysis of variance (ANOVA). The independent variables considered were COVID-19 positivity (COVID+ vs. COVID−) and the position assumed (orthostatism vs. clinostatism). We used the SPSS package (version 20) to perform statistical comparisons. For all the analyses performed, statistical significance was settled at *p* < 0.05.

## 3. Results

A total of 20 subjects were enrolled in the COVID+ group. No significant differences were observed between COVID+ and COVID− groups in sex (COVID+: 14/20 (70%) vs. COVID−: 13/20 (65%); χ^2^ = 0.114, *p* = 0.736), age (COVID+: 56.05 ± 19.15, U-test = 229.000, *p* = 0.433), and BMI (COVID+: 25.75 ± 3.86, U-test = 184.000, *p* = 0.664). 

In particular, none of the subjects included in the study presented symptoms or signs of peripheral neuropathy at neurological clinical evaluation in terms of subjective or objective sensory alterations, reduction of deep tendon reflexes, or segmental muscular weakness.

A detailed representation of the demographic and clinical characteristics of the study cohort is available in Table 1.

In a univariate analysis, no significant differences were found regarding the type of drugs assumed and comorbidities between groups.

Pharmacological treatments assumed by COVID+ and COVID− members able to alter the ANS included antidepressants, beta-blockers, ACE inhibitors, sartans, calcium channel blockers, and alpha-1 blockers. Concomitant pharmacological treatments of both groups are described in Table 2.

### 3.1. Pupillometry

COVID+ members showed a higher DV [COVID+: 1.1 (1.0–1.3); COVID−: 0.9 (0.8–1.2); U-test = 276.000, *p* = 0.040], ACA [COVID+: 1.4 (1.1–1.9); COVID−: 1.0 (0.9–1.5); U-test = 277.000, *p* = 0.037], CI [COVID+: 34.4 (30.3–37.3); COVID−: 30.5 (25.0–34.0); U-test = 276.00, *p* = 0.040], and BPD [COVID+: 4.2 (3.6–4.7);COVID−: 3.7 (3.1–4.1); U-test = 276.500, *p* = 0.039]. No significant differences were observed in other AP parameters. Extended results of the AP analysis are available in Table 3.

### 3.2. Sudoscan

No significant differences were observed in ESC distribution between COVID+ and COVID− group. Performing a dichotomization of ESC values referring to the selected cut-offs, we found a higher rate of sudomotor dysfunction (ESC < 70 μS) of the mean feet values in COVID+ than COVID− group (COVID+: 9 (45%); COVID−: 3 (15%); χ^2^ = 4.286, *p* = 0.038). No significant differences were observed in the prevalence of hands sudomotor dysfunction between groups. For details, refer to Table 4.

### 3.3. Heart Rate Variability

No significant differences were observed between COVID+ and COVID− groups in all the parameters analyzed for all the conditions considered (i.e., clinostatism and orthostatism).

### 3.4. Pulse Transit Time

No significant differences were observed between COVID+ and COVID− groups in PTTs distribution for all the conditions considered (i.e., clinostatism and orthostatism).

## 4. Discussion

In this study we observed significant differences in Sudoscan and AP parameters between COVID+ and COVID− subjects, while no differences were found in HRV data and PTT between groups.

In particular, COVID+ patients presented, in AP measurements, higher values of BPD, DV, ACA, and CI. In the Sudoscan evaluation, COVID+ patients presented a higher prevalence of feet sudomotor dysfunction compared to controls. These findings suggest the presence of a complex alteration involving both the sympathetic and parasympathetic branches of ANS. In fact while Sudoscan is an indirect index of sympathetic functioning, AP suggests both sympathetic (DV, BPD) and parasympathetic (ACA, CI, BPD) involvment. To date, there are no data on the possible dysautonomic involvement in not crtitically-ill COVID-19 patients evaluated through the study of PLR dynamics or sudomotor function. The scarce evidence available on AP concern critically-ill patients, in which parameters could be altered by the severity of the illness [37], or patients recovering from COVID-19. Moreover, Sudoscan has been employed in only one study on patients recovered from SARS-CoV-2 infection.

Concerning critically-ill COVID-19 patients, a study conducted by Vrettou et al. [38] on 18 members with respiratory failure requiring mechanical ventilation for >48 h did not find, after a statistical correction for possible confounders (i.e., sedation), significant differences in PLR dynamics between SARS-CoV-2 infected patients and people suffering respiratory failure from other causes. In another study, Battaglini et al. [39] found an altered pupillary reactivity in about 31% of patients admitted to the intensive care unit (ICU) for severe COVID-19, correlating this finding to the increase of intracranial pressure. Regarding the post-COVID evidence, Karahan et al. [40] observed, one month after healing from the disease, a higher BPD and CV, and lower values of dilatation latency and duration of pupil constriction than healthy controls. 

To date, sudomotor function has been investigated in only one study conducted in patients presenting at least one neurological symptom within 3 months from acute infection. Occurrence of feet sudomotor dysfunction, defined as an ESC < 70 μS, was found in 26% of the study population, mostly in older patients subjected to previous antiviral treatment. Similarly to our study, no significant differences were reported regarding the upper limb ESC [41].

In this study we did not find any correlation between COVID-19 and changes in HRV or PTT parameters.

In the framework of COVID-19 related dysautonomia, a possible interaction between SARS-CoV-2 infection and HRV has already been investigated by several authors.

Aragòn-Benedì et al. [42] found a predominance of the parasympathetic tone, and, at the same time, a withdrawal of the sympathetic activity with an overall reduction of HRV in 14 ICU-admitted COVID-19 patients, more prominent in people who presented a worse outcome. This finding was interpreted by the author as a consequence of the pathological, cholinergic, anti-inflammatory response which follows the initial sympathetic overactivity, inducing immune anergy and worse outcome. A predominance of sympathetic activity during the first phase of the infection has been reported by Pan et al. [43], who found in critically-ill patients an overall reduction of HRV, in terms of SDNN, SDANN, and an increase in the LF/HF, correlated with humoral biomarker increases such as NT-proBNP and D-dimer. In keeping with this, Hasty et al. [44] found, in 16 mild COVID-19 patients, an overall decrease in HRV parameters which anticipated by almost 72 h a spike in the level of C-reactive protein (CRP). These contrasting results could be explained by the sympathetic activation which occurs during the development and exacerbation of the systemic inflammatory response syndrome (SIRS) in severe forms of the disease. Yet once SIRS is established, the sympathetic-vagal balance could shift towards a parasympathetic system predominance, trying to taper down the systemic inflammation [45]. Regarding the effects of SARS-CoV-2 on peripheral vessels, only few studies have been conducted, all demonstrating an increased arterial stiffness in acute COVID-19 patients [46,47]. Moreover, the arterial stiffness increase seems to correlate with outcome measures, such as mortality and length of hospitalization [47]. In our study, we did not observe significant differences in either HRV and PTT parameters between case and controls. The lack of significant difference regarding HRV and PTT in our study could be related to the low sensitivity of the methods in not critically-ill COVID-19 patients.

Furthermore, the lack of statistical significance with these methods could also be linked to the small sample size or to the disease phase at the moment of registration.

In any case, our results confirm the presence of autonomic disfunction encompassing both sympathetic and parasympathetic system.

This is the first study to investigate the presence of sudomotor dysfunction in the acute phase of the disease, and the first to analyze PLR through AP in not critically-ill patients with an active form of COVID-19. All these results suggest that dysautonomia could significantly contribute to the spectrum of COVID-19-related neurological disorders in the acute phase of the disease. The quick recognition of these clinical features could ameliorate the in-hospital management of these patients.

Even if we cannot determine the origin of SARS-CoV-2-related AD, the absence of symptoms and signs of peripheral neuropathy in our study sample suggest an involvement of the central component of the ANS. 

The main limitation of the study is the small sample size, which makes it difficult to extend the results to the general SARS-CoV-2 affected population. Another limitation is the concomitant use, in some patients, of pharmacological treatments which could alter ANS functioning, such as Sartans [48]. Moreover, we selected a control group of healthy subjects with no ongoing infections; therefore, we cannot exclude the role of the inflammatory state in inducing AD. Finally, we cannot exclude measurement errors due to the single measurements performed for each patients in order to reduce the exposure time of the operators to SARS-CoV-2. Further studies, with larger sample size, are needed.

## 5. Conclusions

In this study we found an alteration in Sudoscan and pupillometry parameters, in acute COVID-19, suggesting already the presence of AD in the acute phase of the disease. On the other hand, no significant findings were observed in PTT and HRV analysis. Further studies investigating the origin of the SARS-CoV-2-related AD trough an instrumental evaluation, such as cardiac Iodine-123 metaiodobenzyl-guanidine scintigraphy are needed.

## Figures and Tables

**Table 1:** Clinical and demographic feature of COVID+ and COVID− groups. Categorical variables are expressed as number (*n*) and percentage (%). Numerical variables are expressed as mean ± standard deviation, since they showed a normal distribution in a normality test (i.e., Shapiro-Wilk test).

Clinical Features		COVID+ (*n* = 20)	COVID− (*n* = 20)
Male sex	*n* (%)	14 (70%)	13 (65%)
Age—years	Mean ± SD	56.05 ± 19.15	52.55 ± 13.71
BMI	Mean ± SD	25.75 ± 3.86	26.40 ± 3.39
**COVID symptoms**			
Dyspnoea	*n* (%)	15 (75%)	0 (0%)
Fever at time of evaluation	*n* (%)	0 (0%)	0 (0%)
Diarrhoea	*n* (%)	10 (50%)	3 (15%)
Dizziness	*n* (%)	12 (60%)	1 (5%)
Pneumonia	*n* (%)	14 (70%)	0 (0%)
**∆ symptoms onset-registration time**	Median (IQR)	6 (4–8)	
**Comorbidities**			
Hypertension	*n* (%)	10 (50%)	7 (35%)
Heart disease	*n* (%)	4 (20%)	1 (5%)
Dysthyroidism	*n* (%)	1 (5%)	2 (10%)
Renal failure	*n* (%)	1 (5%)	0 (0%)
Ocular disease	*n* (%)	0 (0%)	2 (10%)

**Table 2:** Concomitant pharmacological treatments of COVID+ and COVID− groups. Variables are expressed as number (*n*) and percentage (%).

Pharmacological Treatments	COVID+ (*n* = 20)	COVID− (*n* = 20)
α-blockers, *n* (%)	0 (0%)	1 (5%)
β-blockers, *n* (%)	5 (25%)	3 (15%)
ACE-inhibitors, *n* (%)	1 (5%)	2 (10%)
Sartans, *n* (%)	6 (30%)	1 (5%)
Calcium channel blockers, *n* (%)	3 (15%)	1 (5%)
Antiarrhythmics, *n* (%)	0 (0%)	0 (0%)
Antiepileptic drugs, *n* (%)	0 (0%)	1 (5%)
Antidepressants, *n* (%)	1 (5%)	0 (0%)
Antipsychotics, *n* (%)	1 (5%)	0 (0%)
Hypnotic drugs, *n* (%)	2 (10%)	3 (15%)

**Table 3:** Results of automated pupillometry analysis. Categorical variables are expressed as number (*n*) and percentage (%), and numerical variable as median and interquartile range (IQR). For categorical variables, we adopted the Pearson’s χ^2^ test. Numerical variables were analyzed by means of Mann-Whitney U-test. Significance was set at *p* < 0.05. We can see highlighted the results that reached statistical significance.

		COVID+ (*n* = 20)	COVID− (*n* = 20)	Mann-Whitney		Pearson’s χ^2^	
**PUPILLOMETRY**				**U-Test**	***p***	**Test**	***p***
**Sympathetic parameters**							
Dilatation velocity (mm/s)	Median (IQR)	1.1 (1.0–1.3)	0.9 (0.8–1.2)	**276.000**	**0.040**		
**Parasympathetic parameters**							
Reflex latency (ms)	Median (IQR)	240 (215–250)	233 (211–250)	220.000	0.581		
Constriction velocity (mm/s)	Median (IQR)	2.3 (1.9–2.7)	2.1 (1.7–2.7)	250.00	0.176		
Maximum constriction velocity (mm/s)	Median (IQR)	3.7 (3.2–4.4)	3.1 (2.8–4.4)	241.000	0.267		
Minimum pupil diameter (mm)	Median (IQR)	2.8 (2.5–3.1)	2.6 (2.2–2.7)	259.500	0.107		
Absolute constriction amplitude (mm)	Median (IQR)	1.4 (1.1–1.9)	1.0 (0.9–1.5)	**277.000**	**0.037**		
Constriction index (%)	Median (IQR)	34.0 (30.3–37.3)	30.5 (25.0–34.0)	**276.000**	**0.040**		
**Mixed parameters**							
Baseline pupil diameter (mm)	Median (IQR)	4.2 (3.6–4.7)	3.7 (3.1–4.1)	**276.500**	**0.039**		
Neuro-pupillary index	Median (IQR)	4.4 (4.1–4.6)	4.5 (4.2–4.6)	172.000	0.445		
Neuro-pupillary index < 3	*n* (%)	0 (0%)	1 (5%)			1.026	0.500

**Table 4:** Results of Sudoscan analysis. Categorical variables are expressed as number (*n*) and percentage (%), and numerical variables as mean ± SD, since they were normally distributed. For categorical variables, we adopted the Pearson’s χ^2^ test. Numerical variables were analyzed by means of *t*-test. Significance was set at *p* < 0.05. We can see highlighted the results that reached statistical significance.

		COVID+ (*n* = 20)	COVID− (*n* = 20)	*t*-Test		Pearson’s χ^2^	
**SUDOSCAN**				***t*** **-Test**	***p***	**Test**	***p***
**Hands**							
Mean ESC hands	mean ± SD	66.5 ± 17.3	70.3 ± 13.4	0.767	0.448		
Mean ESC right hand	mean ± SD	66.8 ± 17.4	69.1 ± 12.9	0.485	0.631		
Mean ESC left hand	mean ± SD	66.6 ± 17.9	68.9 ± 14.6	0.437	0.665		
**Sudomotor impairment**							
ESC hands < 60 mcs	*n* (%)	6 (30%)	5 (25%)			0.125	0.723
ESC right hand < 60 mcs	*n* (%)	6 (30%)	4 (20%)			0.533	0.465
ESC left hand < 60 mcs	*n* (%)	7 (35%)	6 (30%)			0.114	0.736
**Feet**							
Mean ESC feet	mean ± SD	67.5 ± 22.3	76.8 ± 11.8	1.647	0.108		
Mean ESC right foot	mean ± SD	68.8 ± 21.9	75.0 ± 19.2	0.945	0.351		
Mean ESC left foot	mean ± SD	69.0 ± 23.9	76.8 ± 12.0	1.294	0.204		
**Sudomotor impairment**							
ESC feet < 70 mcs	*n* (%)	9 (45%)	3 (15%)			**4.286**	**0.038**
ESC right foot < 70 mcs	*n* (%)	8 (40%)	4 (20%)			1.905	0.168
ESC left foot < 70 mcs	*n* (%)	8 (40%)	3 (15%)			3.135	0.077

## Data Availability

The data that support the findings of this study are available from the corresponding author upon reasonable request.
